# *Setaria*: A Food Crop and Translational Research Model for C_4_ Grasses

**DOI:** 10.3389/fpls.2016.01885

**Published:** 2016-12-15

**Authors:** Shankar R. Pant, Sonia Irigoyen, Andrew N. Doust, Karen-Beth G. Scholthof, Kranthi K. Mandadi

**Affiliations:** ^1^Texas A&M AgriLife Research and Extension Center, Texas A&M University SystemWeslaco, TX, USA; ^2^Department of Plant Biology, Ecology, and Evolution, Oklahoma State UniversityStillwater, OK, USA; ^3^Department of Plant Pathology and Microbiology, Texas A&M UniversityCollege Station, TX, USA

**Keywords:** *Setaria*, foxtail millet, green foxtail, C_4_ grasses, nutrition, genomics, biotic and abiotic stress, model systems

It is increasingly evident that new environmentally sustainable agricultural practices will be necessary to support an estimated human population of 9 billion by 2050. Considering the predicted climate change-associated events such as flooding, drought, increased temperatures, and increased soil salinity, it is critical to broaden our study and use of cereals and small grains. Millets offer significant potential toward meeting these goals. The genus *Setaria* comprises more than one hundred species distributed in a wide range of habitats in Asia, Africa, South America, and North America. Millets, including foxtail millet (*Setaria italica*), pearl millet (*Pennisetum glaucum*) and proso millet (*Panicum milaceum*), are consumed by millions of humans across the world, as well as used as forage and bird feed. Recently, foxtail millet and its wild-progenitor, green foxtail millet (*S. viridis*), have emerged as model plant systems for studying C_4_ grass biology and other agronomic traits (Doust et al., 2009; Li and Brutnell, 2011; Lata et al., 2013; Mandadi et al., 2014; Xianmin et al., 2014; Brutnell, 2015; Brutnell et al., 2015). Here, we comment on the significance of *S. italica* and *S. viridis* as model systems for agronomic and translational research related to improvement of nutritional quality, biotic and abiotic stress responses, photosynthetic efficiency and biomass potential of C_4_ grasses.

Foxtail millet is in the *Paniceae* tribe (sub-family Panicoideae of the *Poaceae*), which also contains pearl millet and proso millet. Foxtail millet was domesticated from green foxtail in northern China ca. 8000 years ago (Barton et al., 2009), and has been widely cultivated in arid and semi-arid regions of Asia, Africa and the Americas (Lata et al., 2013). It is one of the most resilient cereal crops, with good yields in dry and marginal land with minimal agricultural inputs. Foxtail millet also adapts well to adverse weather conditions such as low and unpredictable precipitation (Lata et al., 2013; Muthamilarasan and Prasad, 2015). Furthermore, it appears to be resilient to multiple abiotic and biotic stresses such as drought (Zhang et al., 2005; Meng et al., 2009; Lata et al., 2011), salinity (Ardie et al., 2015) and fungal diseases (Xu et al., 2011). Because global climate change will have significant adverse effects on the production of other major cereals, foxtail-, pearl- and proso-millets are increasingly attractive alternatives for small grain production (Tadele, 2016).

Green foxtail millet has also come into the spotlight in recent years as an attractive alternative to more established grass model systems such as rice and maize (Figure 1). *S. viridis* offers several research advantages, including short stature (10–30 cm), rapid life cycle (6–9 weeks), prolific seed production (~13,000 seeds per plant), self-compatibility, small genome size (~395 Mb), diploid genetics (2*n* = 18), ability to be grown in controlled environments under relatively low light levels, and amenability to transformation (Devos et al., 1998; Doust et al., 2009; Brutnell et al., 2010; Li and Brutnell, 2011; Wang et al., 2011; Xianmin et al., 2014; Saha and Blumwald, 2016). Like maize, green and foxtail millets are C_4_ plants, but they have smaller genomes and are true diploids. In combination with sorghum (*Sorghum bicolor*), *S. italica* and *S. viridis* are proving valuable in studying C_4_ photosynthesis, with the long-term goal of engineering C_4_ traits into important C_3_ crops such as rice and wheat.

**Figure 1:**
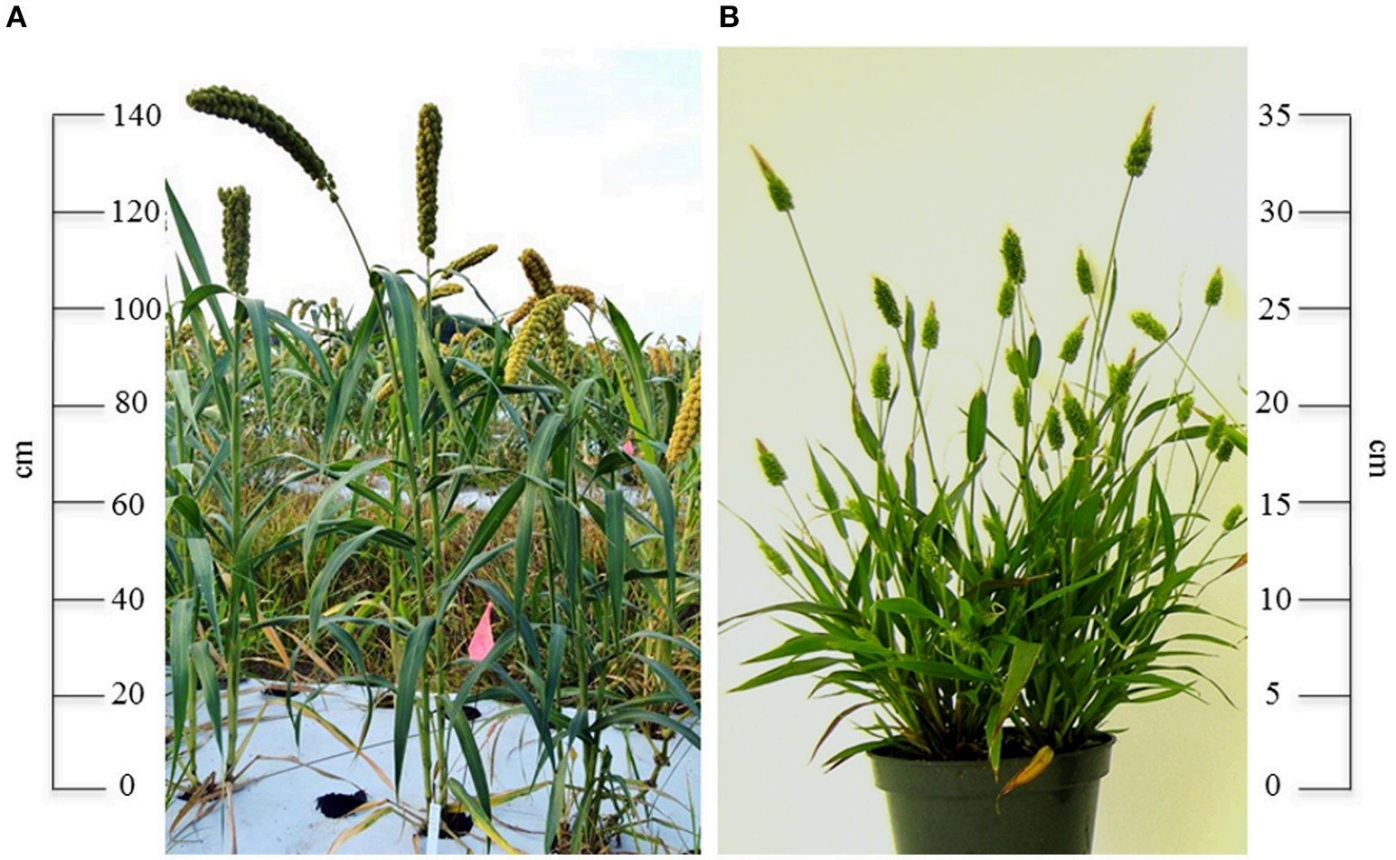
**(A)** *Setaria italica* and **(B)** *S. viridis* are emerging model species for millet and C_4_ grass biology. Photo credits: John Hodge and Jesse D. Pyle.

The *S. italica* and *S. viridis* system offer many genetic resources, including sequenced draft genomes (Bennetzen et al., 2012; Zhang et al., 2012) (*Setaria viridis* v1.1, DOE-JGI, http://phytozome.jgi.doe.gov) and a high-density haplotype map of genome variation (Jia et al., 2013). Comparisons of the arrangement of the nine chromosomes of *S. italica* and *S. viridis* with the corresponding chromosomes of rice and sorghum reveal relatively few rearrangements, and the genome sequence has been used to guide the assembly of the polyploid genome of its close relative, *Panicum virgatum* (switchgrass), a promising bioenergy feedstock (Daverdin et al., 2015). The high-quality genome sequence of foxtail millet has also allowed in-depth analyses of transposon family dynamics and locations that reveal hitherto unsuspected variation between transposon families in insertion site preference and turnover (Bennetzen et al., 2016). Mutant populations have been characterized in both foxtail millet and green foxtail millet, and the identity of candidate genes was revealed by novel high-throughput sequencing approaches (Li et al., 2016; Liu et al., 2016; Martins et al., 2016; Xue et al., 2016). *S. italica* and *S. viridis* have been used in the characterization of important agronomic traits, including yield-related architectural traits such as height, branching, biomass, flowering time and domestication-related traits such as shattering (Qian et al., 2012; Jia et al., 2013; Mauro-Herrera et al., 2013; Wang et al., 2013; Doust et al., 2014; Gupta et al., 2014; Layton and Kellogg, 2014; Qie et al., 2014; Fahlgren et al., 2015; Fang et al., 2016; Hodge and Kellogg, 2016; Liu et al., 2016; Mauro-Herrera and Doust, 2016).

In plants, salicylic acid (SA), jasmonic acid (JA) and ethylene (ET) are key defense hormones that mediate signaling during plant-microbe interaction. Prevailing evidence, primarily based on studies of dicot plants, suggest that SA and JA/ET pathways are antagonistic to each other during plant-microbe interactions. Recently, in conjunction with *Brachypodium distachyon* (a C_3_ grass), *S. viridis* was used to characterize the dynamics of C_3_ vs. C_4_ plant defense signaling responses against a diverse group of grass-infecting viruses (Scholthof, 1999; Mandadi and Scholthof, 2012; Mandadi et al., 2014). This study showed that SA and JA/ET crosstalk does exist in *S. viridis* during grass-virus interactions; however, there were also unique C_4_ host-dependent signaling responses. *S. italica* was also used to dissect grass defense signaling pathways modulated during an incompatible interaction with a fungal pathogen, *Uromyces setariae-italicae* (Li et al., 2015). Together, these studies demonstrate the utility of *S. italica* and *S. viridis* for fundamental research pertaining to plant-microbe interactions.

*S. italica* and *S. viridis* are also very useful for translational research related to bioenergy traits. In addition to plant-derived sugars it has been shown that cell-wall derived cellulose, hemicellulose and lignin are sustainable sources for bioenergy production. Owing to their superior carbon-assimilation and photosynthetic pathways, several C_4_ grasses such as switchgrass, *Miscanthus*, and energycane have emerged as feedstocks for plant ligno-cellulosic biomass (Brutnell et al., 2010). Efforts are underway to engineer C_4_ photosynthetic pathways into C_3_ crops (e.g., rice) to improve photosynthetic capacity and yield (von Caemmerer et al., 2012; Karki et al., 2013). However, the genomes and genetics of the leading bioenergy grasses are complex, and are a hindrance in deciphering the mechanisms of C_4_ photosynthesis, and ultimately to engineer them into C_3_ grasses. Both *S. italica* and *S. viridis* are C_4_ grasses and their genomes are highly syntenic to other Panicoid grasses (Kumari et al., 2013) and, in this context, these model plants offer powerful tools to understand the genetics of biomass production of panicoid bioenergy grasses, as well as to limn the evolution of C_4_ vs. C_3_ traits (Doust et al., 2009; Li and Brutnell, 2011; Lata et al., 2013; Mandadi et al., 2014; Brutnell et al., 2015; Muthamilarasan and Prasad, 2015; Martin et al., 2016).

In addition to enabling research into agronomic traits, *S. italica* and *S. viridis* are valuable models in the study of phytonutrient pathways pertaining to small grain millets. For example, *S. italica* is a good source of calories and essential micronutrients, and has higher nutritive value than major cereal grains like rice, wheat and sorghum. *S. italica* also contains high levels of proteins, dietary fibers, vitamins, anti-oxidants and non-starchy polysaccharides with low glycemic index, when compared to rice, wheat and sorghum (Taylor et al., 2006; Suma and Urooj, 2012; Amadou et al., 2013; Muthamilarasan and Prasad, 2015; Muthamilarasan et al., 2015). Moreover, over fifty phenolic compounds belonging to classes such as hydroxybenzoic acids, hydroxycinnamic acids and flavonoids were identified from *S. italica* (Chandrasekara and Shahidi, 2011b). In *in vitro* studies, phenolic compounds derived from *S. italica*, exhibited health promoting properties including antioxidant and free radical quenching, which helps in boosting immunity and inhibiting cancer cell proliferation (Dykes and Rooney, 2006; Chandrasekara and Shahidi, 2010, 2011a,b, 2012; Muthamilarasan and Prasad, 2015). Notwithstanding these reported nutritional qualities, certain anti-nutritional constraints were also reported for *S. italica*. These include the presence of toxic substances, some of which might be harmful to human health. For instance, some foxtail millet grains contain goitrogens that suppress thyroid activity which may have a role in goiter—an enlargement of the thyroid gland (Gaitan et al., 1989; Gluchoff-Fiasson et al., 1989; Elnour et al., 2000). The major goitrogenic and antithyroid compounds isolated were C-glycosylflavones (Gaitan et al., 1989; Gluchoff-Fiasson et al., 1989). Furthermore, *in vivo* experiments with vitexin (one of the three major C-glycosylflavones) in rats revealed evidence of antithyroid activity (Gaitan et al., 1995). In this context, further research is needed to carefully delineate the nutritional and anti-nutritional properties of phytochemicals present in *S. italica* and perhaps other small grain millets. The availability of *S. italica* and *S. viridis* genomic and genetic resources should enable research into identifying and inactivating such anti-nutritional compounds using novel genome-editing technologies (e.g., CRISPR-CAS9) to enhance *S. italica* nutritional traits.

Given the importance of *S. italica* as a food crop and in the study of grass biology, we suggest that *S. italica*, and its wild-progenitor, *S. viridis*, are promising translational research models to study nutritional pathways, abiotic and biotic stress resistance pathways, as well as to advance C_4_ photosynthesis and bioenergy research.

## Author contributions

SP, SI, AD, KS, and KM contributed to the design, preparation, and editing of the manuscript.

## Funding

This manuscript was supported by funds from USDA-NIFA-AFRI (2016-67013-24738) to KS and KM, Texas A&M AgriLife Research Bioenergy/Bioproducts Grant (124738-96210) to KM, and NSF Plant Genome (IOS-1339332) to AD.

### Conflict of interest statement

The authors declare that the research was conducted in the absence of any commercial or financial relationships that could be construed as a potential conflict of interest.
